# Use of Videos Improves Informed Consent Comprehension in Web-Based Surveys Among Internet-Using Men Who Have Sex With Men: A Randomized Controlled Trial

**DOI:** 10.2196/jmir.6710

**Published:** 2017-03-06

**Authors:** Eric William Hall, Travis H Sanchez, Aryeh D Stein, Rob Stephenson, Maria Zlotorzynska, Robert Craig Sineath, Patrick S Sullivan

**Affiliations:** ^1^ Department of Epidemiology Rollins School of Public Health Emory University Atlanta, GA United States; ^2^ Hubert Department of Global Health Rollins School of Public Health Emory University Atlanta, GA United States; ^3^ Department of Health Behavior and Biological Sciences School of Nursing and The Center for Sexuality and Health Disparities University of Michigan Ann Arbor, GA United States

**Keywords:** informed consent, surveys and questionnaires, HIV

## Abstract

**Background:**

Web-based surveys are increasingly used to capture data essential for human immunodeficiency virus (HIV) prevention research. However, there are challenges in ensuring the informed consent of Web-based research participants.

**Objective:**

The aim of our study was to develop and assess the efficacy of alternative methods of administering informed consent in Web-based HIV research with men who have sex with men (MSM).

**Methods:**

From July to September 2014, paid advertisements on Facebook were used to recruit adult MSM living in the United States for a Web-based survey about risk and preventive behaviors. Participants were randomized to one of the 4 methods of delivering informed consent: a professionally produced video, a study staff-produced video, a frequently asked questions (FAQs) text page, and a standard informed consent text page. Following the behavior survey, participants answered 15 questions about comprehension of consent information. Correct responses to each question were given a score of 1, for a total possible scale score of 15. General linear regression and post-hoc Tukey comparisons were used to assess difference (*P*<.001) in mean consent comprehension scores. A mediation analysis was used to examine the relationship between time spent on consent page and consent comprehension.

**Results:**

Of the 665 MSM participants who completed the comprehension questions, 24.2% (161/665) received the standard consent, 27.1% (180/665) received the FAQ consent, 26.8% (178/665) received the professional consent video, and 22.0% (146/665) received the staff video. The overall average consent comprehension score was 6.28 (SD=2.89). The average consent comprehension score differed significantly across consent type (*P*<.001), age (*P*=.04), race or ethnicity (*P*<.001), and highest level of education (*P*=.001). Compared with those who received the standard consent, comprehension was significantly higher for participants who received the professional video consent (score increase=1.79; 95% CI 1.02-2.55) and participants who received the staff video consent (score increase=1.79; 95% CI 0.99-2.59). There was no significant difference in comprehension for those who received the FAQ consent. Participants spent more time on the 2 video consents (staff video median time=117 seconds; professional video median time=115 seconds) than the FAQ (median=21 seconds) and standard consents (median=37 seconds). Mediation analysis showed that though time spent on the consent page was partially responsible for some of the differences in comprehension, the direct effects of the professional video (score increase=0.93; 95% CI 0.39-1.48) and the staff-produced video (score increase=0.99; 95% CI 0.42-1.56) were still significant.

**Conclusions:**

Video-based consent methods improve consent comprehension of MSM participating in a Web-based HIV behavioral survey. This effect may be partially mediated through increased time spent reviewing the consent material; however, the video consent may still be superior to standard consent in improving participant comprehension of key study facts.

**Trail Registration:**

Clinicaltrials.gov NCT02139566; https://clinicaltrials.gov/ct2/show/NCT02139566 (Archived by WebCite at http://www.webcitation.org/6oRnL261N).

## Introduction

Men who have sex with men (MSM) continue to be the group most impacted by human immunodeficiency virus (HIV) in the United States. In 2014, 70% of all new infections in the United States occurred among MSM [1]. In many regions around the world, HIV incidence rates among MSM have been increasing [2-4]. This increase has been theorized, in part, to result from an increase of MSM using the Internet to facilitate sexual relationships through partner selection websites [4-7]. There is evidence that men who arrange Web-based sexual encounters may have increased odds of having unprotected anal intercourse [5,6,8], which presents an increased risk for HIV transmission. As a result, Web-based HIV research and prevention opportunities can be particularly valuable.

Increasing Internet accessibility also can improve the delivery of health services among all MSM. Internet usage is highest among young Americans: 96% of 18-29 year olds used the Internet in 2015 [9]. This high coverage provides an opportunity to deliver Web-based HIV prevention and treatment information on a large scale [10,11]. However, HIV prevention research is first needed to establish the efficacy of prevention programs.

The Internet is increasingly being used to recruit underserved MSM and engage them in HIV prevention research [7]. Compared with face-to-face interviews, Web-based research can collect data from a large number of people in a short period of time [12]. Web-based studies can anonymously include MSM who might be stigmatized if their sexual interests were known publicly [13]. Furthermore, Web-based research can provide access to men who might be at an elevated risk of HIV infection. Compared with participants recruited at physical venues, MSM recruited through the Web are more likely to self-report sexually transmitted infections (STIs), anal intercourse, and unprotected anal intercourse [14-18].

However, there are challenges to using the Internet for HIV prevention research [19]. The data can be subject to selection bias because of demographic differences in Internet use and access [9]. Willingness to click on an advertisement, provide consent, and begin a Web-based survey differs by age, race, education, and urbanicity of residence [20]. Furthermore, there are ethical challenges that are unique to Web-based research [11,21]. When consent is administered on the Web, it is difficult to confirm the age, competency, and comprehension of a potential participant [22]. This is particularly concerning because many of the topics that are typically covered in HIV prevention research (sexual behavior, drug use, commercial sex work, and so on) contain confidential and sensitive information.

Most Web-based consent processes involve the respondent reading a document and then indicating that they agree to participate by clicking a button. This is similar to the lengthy terms-of-service documents that many Internet and mobile phone services require, and participants may develop a habit of accepting documents without fully reviewing them. An ethical review of a Web-based HIV prevention study called for innovative ways to provide informed consent to participants [23]. Better alignment between a participant’s typical Web-based experience and the informed consent process may improve their consumption of Web-based consent information.

Many Web-based social media interactions now involve the use of photos and videos [24]. Internet users may also be more acclimated to list-based or brief summaries when they do choose to consume textual information. Previous Web-based research with MSM has also shown that brief consent summaries may improve consent information comprehension over a standard consent process, but this research was done before Web-based interactions became more photo- or video-focused and therefore we did not examine this alternative [19,25]. The objective of our study was to assess if comprehension of key informed consent facts improved through the use of these new techniques versus a standard text-based informed consent form.

## Methods

### Study Design

**Figure 1:**
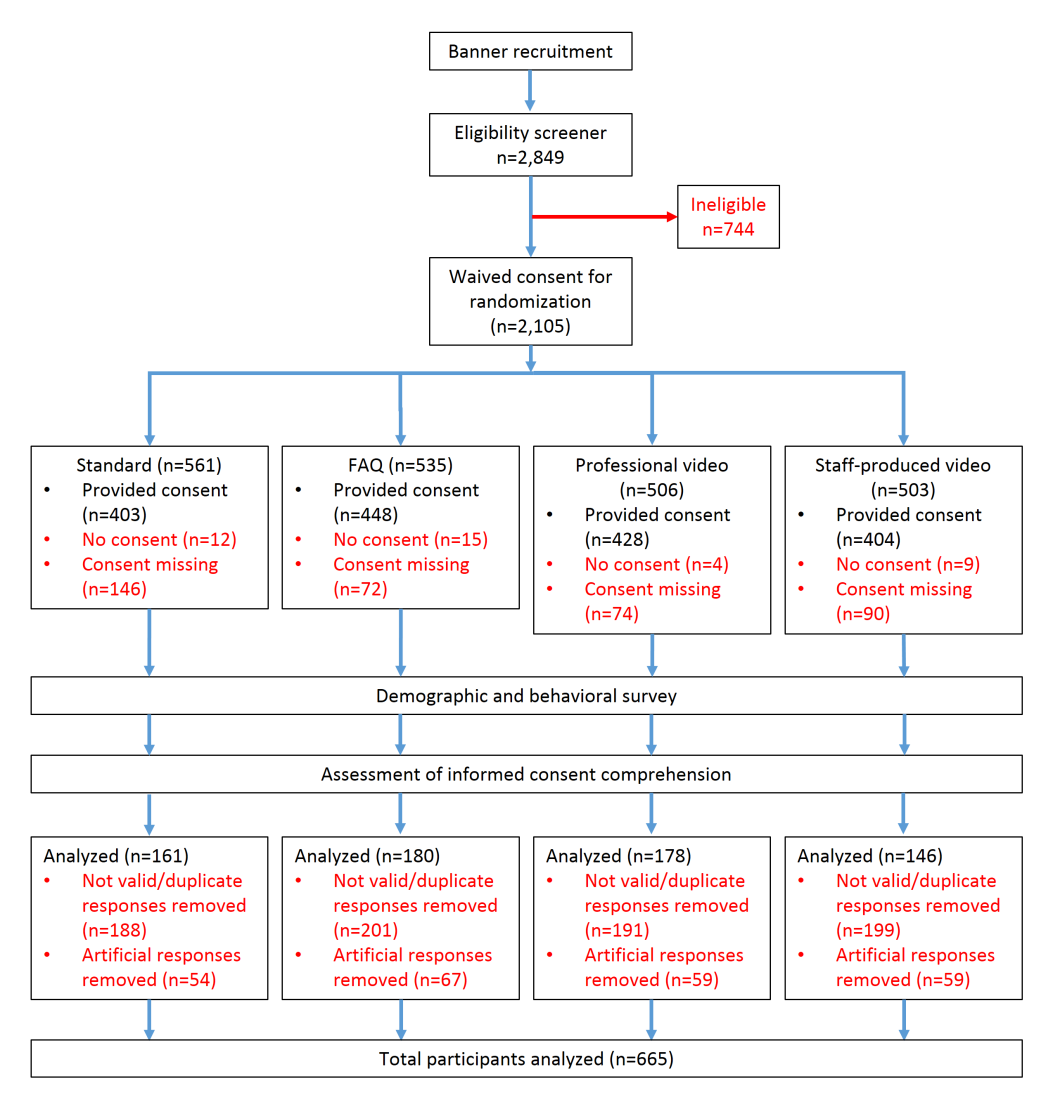
Consort diagram and study scheme for participant enrollment of men who have sex with men in a randomized trial of informed consent methods, United States, 2014.

A randomized trial (Clinicaltrials.gov NCT02139566) was conducted to assess alternative methods of administering informed consent in a Web-based HIV behavioral research study with young adult MSM residing in the United States. All survey data were collected through a Health Insurance Portability and Accountability Act (HIPAA)–compliant Web-based survey platform (SurveyGizmo, Boulder, CO). Participants were recruited from Facebook from July 28 to September 8, 2014. Banner advertisements were presented to men who indicated they were interested in media and services targeted toward gay men or interested in men on their Facebook profiles. Respondents who clicked on the banners were taken to an eligibility screener. To be eligible, respondents had to report that they lived in the United States, were 18 to 34 years old, were male at birth, and have had sex with another man in the past 12 months. Eligible respondents were randomized by the survey into one of the 4 consent groups: standard informed consent, frequently asked questions (FAQs) informed consent, a video informed consent produced by a professional company (Tom Coggia, iconAtomic), or a video informed consent produced by study staff (Figure 1). The 3 alternative consent methods were developed based on the results of 5 online focus groups which engaged MSM in discussions about how they considered and interacted with Web-based informed consent documents.

The standard informed consent consisted of a consent document presented in a scrolling window. The FAQ informed consent consisted of 19 questions (e.g., “What is the purpose of the study?”,“Who is conducting the study?”) that revealed short paragraphs of information when the participant clicked on them (Multimedia Appendix 1). The 2 video consents were each about 3.5 minutes long and covered the same topics as the standard and FAQ consents, but used a different script written by the researchers and were presented verbally with bullets of key points highlighted on the screen (Multimedia Appendices 2 and 3). The level of reading comprehension was consistent between the video scripts, the standard consent, and the FAQ consent. All respondents also had the option to open or download the full standard informed consent document as a portable document format (PDF). This study was reviewed and approved by Emory University Institutional Review Board that approved a waiver of written informed consent. After providing consent, participants were directed to a 15-minute Web-based survey on demographic characteristics, sexual history, HIV and STI testing history, and use of HIV prevention services. At the end of the survey, we asked 15 questions about essential elements covered in the informed consent process (Multimedia Appendix 4). Participants did not know there would be questions about the informed consent process. Participants who completed the survey and answered the comprehension questions were given a US $20 Amazon gift card sent by email.

### Measures

The primary analytic outcome was total consent comprehension score, which was calculated by tabulating the number of consent comprehension questions that each participant got correct. Similar to previously used methods, 1 point was given for each correct question for a minimum total score of 0 and maximum of 15 [26]. The 15 consent comprehension questions were selected from a larger pool of 29 possible questions with multiple questions from each of the essential elements of informed consent [27]. Before the main study, consent comprehension questions were pilot-tested among MSM recruited in the same manner as the main study (banner advertisements on Facebook) and were presented with a standard consent form. The pilot study recruited 132 MSM who completed a short survey and the 29 comprehension questions. The data were analyzed for frequencies of correct responses to the 29 pilot consent comprehension questions. At least one question from each of the essential elements was retained. If there were multiple questions from a category, questions with very low or very high correct response rates were removed in order to allow for variability in responses to assess difference in comprehension during the main study.

Time spent on consent was measured by amount of time the respondent stayed on the Web page with the consent information. In order to progress to the next page, respondents had to click on a button indicating they agree (or do not agree) to participate in the study. Demographic variables (race or ethnicity, highest level of education, and age) were summarized as frequencies and percentages in each consent group. Race or ethnicity was coded into 6 categories: Asian, black, Hispanic, multiracial, American Indian or Alaska Native, and white.

### Analyses

All analyses were done in SAS version 9.4 (SAS Institute, Cary, NC). As recommended for Web-based participant recruitment, a deduplication protocol was determined before data collection to remove duplicate or artificial survey attempts [28]. While implementing the study, we noted a pattern of frequent responses from the same Internet protocol (IP) address, very short survey completion times, and unusual email addresses—all indicators of potential fraudulent responses from artificial hacking or bot programs possibly aimed at getting the monetary incentive. We immediately put measures in place to curtail this activity, including Completely Automated Public Turing test to tell Computers and Humans Apart (CAPTCHA) codes and verification of email addresses submitted for incentives [29]. For purposes of analyses, the following types of responses were excluded: a single IP address submitted 5 or more responses, total completed survey time was less than 5 minutes, or invalid email address as determined by staff member reviewing all submitted emails. All analysis was intent-to-treat and all participants were analyzed in the original randomization groups.

Pearson chi-square tests were used to assess demographic differences by consent randomization group. Time spent on consent and consent comprehension score were analyzed as continuous variables and summarized using medians and interquartile ranges (IQRs) or means and standard deviations, where appropriate. Residuals for both continuous variables were gauged for normality by inspecting histograms, boxplots, and quantile-quantile probability plots. As the time spent on consent was not normally distributed, Kruskal-Wallis tests were used to assess differences in time spent on consent by demographic variables. Time spent on consent was log transformed to assess the correlation with consent comprehension score using Pearson correlation coefficient.

Factors associated with consent comprehension score were assessed using general linear regression. Analyses were conducted for consent group, age, race, and highest level of education. Least-squares means and 95% CIs were reported. When overall significant differences were found, pairwise comparisons were considered using the Tukey-Kramer method [30]. A mediation analysis was conducted to determine if time spent on consent was a mediator in the relationship between consent group and consent comprehension score. Direct effects were calculated to determine the change in consent comprehension score when time on consent was held constant. Indirect effects were calculated to determine the change in consent comprehension score when consent group was held constant and time on consent differed by the amount it differed between consent groups. We used methods for mediation analysis with a multicategorical independent variable as described by Hayes et al [31]. Asymmetric bootstrap procedure with 10,000 resamples was used to calculate bias-corrected CIs around the relative indirect effect estimates. All statistical tests were assessed at alpha=.05.

## Results

Of the 2849 survey responses, there were 665 (23.34%, 665/2849) eligible participants who consented to be part of the study and complete the survey and comprehension questions (Figure 1). There were 161 (24.2%, 161/665) participants in the standard consent group, 180 (27.1%, 180/665) in the FAQ group, 178 (26.8%, 178/665) in the professional video group, and 146 (22.0%, 146/665) in the staff video group (Table 1). When subsetting the data to only include survey responses submitted after the artificial hacking or bot was curtailed, the percentage of valid, eligible surveys didn’t substantially differ (data not presented).

**Table 1:** Demographic characteristics of 665 men who have sex with men who participated in a randomized trial of informed consent methods, by informed consent group randomization, United States, 2014.

Demographic characteristics		Consent group					
		n (%)	Standard n (%)	FAQ^a^ n (%)	Professionally produced video n (%)	Staff-produced video n (%)	*P* value^b^
All			161 (24.2)	180 (27.1)	178 (26.8)	146 (22.0)	
**Race or ethnicity**							.13
	Asian or Pacific Islander	24 (3.6)	3 (1.9)	11 (6.2)	4 (2.3)	6 (4.1)	
	Black	22 (3.3)	5 (3.1)	5 (2.8)	10 (5.7)	2 (1.4)	
	Hispanic	147 (22.1)	31 (19.4)	45 (25.4)	39 (22.3)	32 (22.1)	
	Multiracial	26 (3.9)	6 (3.8)	8 (4.5)	8 (4.6)	4 (2.8)	
	American Indian or Alaska Native	8 (1.2)	1 (0.6)	5 (2.8)	0 (0)	2 (1.4)	
	White	430 (64.7)	114 (71.3)	103 (58.2)	114 (65.1)	99 (68.3)	
	Missing	8 (1.2)					
**Highest level of schooling**							.73
	≤ High school	88 (13.3)	18 (11.2)	25 (14.0)	29 (16.4)	16 (11.0)	
	Some college	288 (43.4)	75 (46.6)	73 (40.8)	80 (45.2)	60 (41.1)	
	Bachelor's degree	183 (27.6)	46 (28.6)	52 (29.1)	42 (23.7)	43 (29.5)	
	Graduate or professional	104 (15.7)	22 (13.7)	29 (16.2)	26 (14.7)	27 (18.5)	
	Missing	2					
**Age, in years**							.34
	18-22	164 (24.7)	46 (28.6)	45 (25.0)	47 (26.4)	26 (17.8)	
	23-27	236 (35.5)	57 (35.4)	58 (32.2)	65 (36.5)	56 (38.4)	
	28-34	265 (39.8)	58 (36.0)	77 (42.8)	66 (37.1)	64 (43.8)	
Age in years, median (IQR^c^)		26 (23-29)	25 (22-28)	26 (23-30)	26 (22-29)	26 (24-30)	.10

^a^FAQ: frequently asked questions.

^b^Calculated by Pearson chi-square test for categorical variables and by Kruskal-Wallis test for age as a continuous variable. All tests were assessed with alpha=.05.

^c^IQR: Interquartile range.

Of the participants that reported a race or ethnicity, most identified as white (64.7%, 430/657) or Hispanic (22.1%, 147/657). The median age was 26 years (IQR: 23-39). There were 88 (13.3%, 88/663) participants who reported that they did not receive any schooling past high school. There was not a statistically significant difference in participant race, highest level of education, or age across the 4 randomized consent groups.

**Figure 2:**
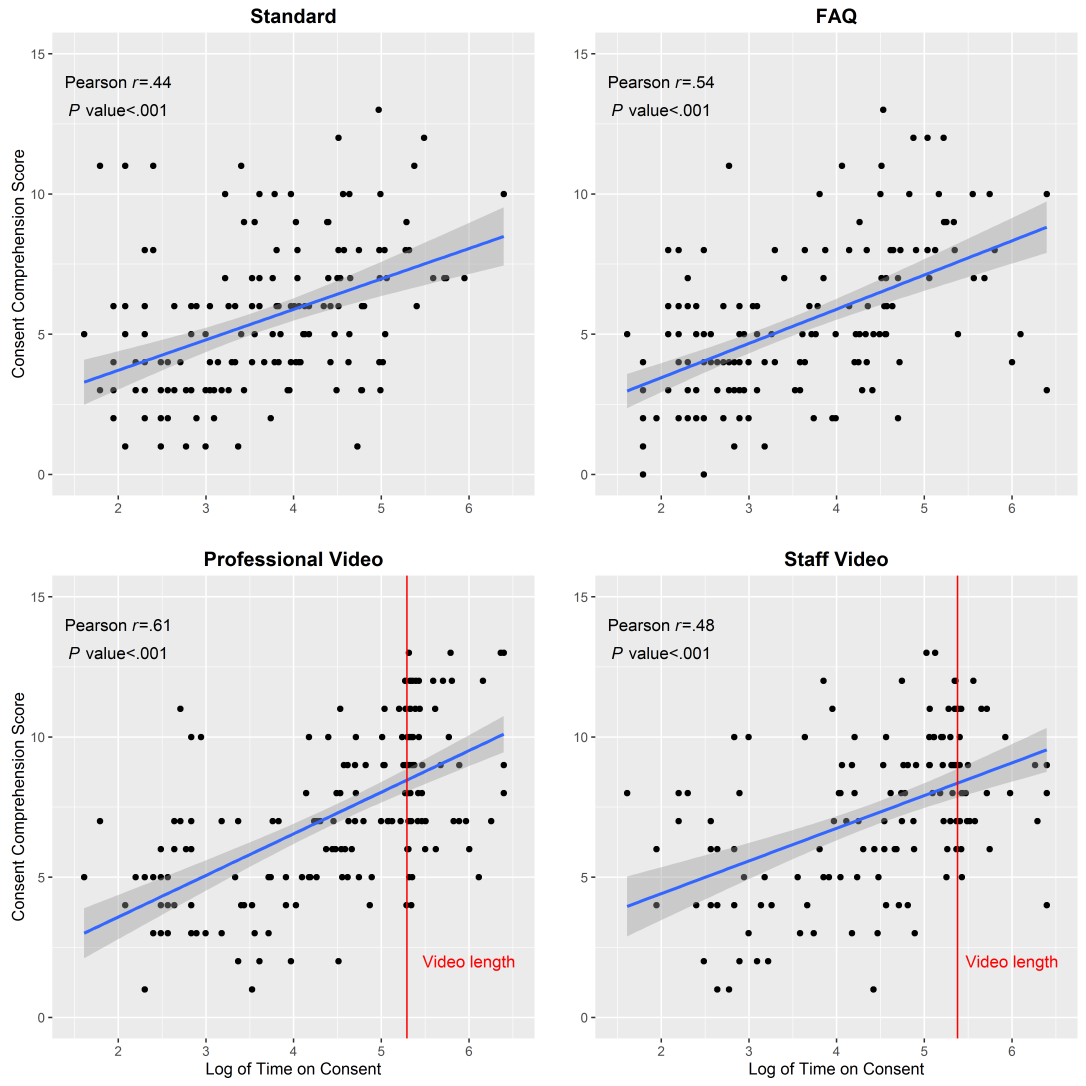
Correlation of time spent on informed consent and consent comprehension score, by type of informed consent, among 665 MSM in a randomized trial of informed consent methods, United States, 2014.

Among all participants, the median time spent on the informed consent was 60.5 seconds (Table 2). The time spent on the consent page significantly differed by type of consent (*P*<.001), with participants in the professional video (median=115.0 seconds, IQR: 37.0-208.0) and staff video (median=117.0 seconds, IQR: 39.0-212.0) consent groups spending more time than participants in the standard (median=37.0 seconds, IQR: 14.5-88.0) and FAQ (median=20.5 seconds, IQR: 11.0-82.0) consent groups. About 36.5% (65/178) of participants in the professional video group and 19.9% (29/146) of participants in the staff video group stayed on the consent page for an amount of time that was longer than the duration of the video (professional video: 200 seconds; staff video: 218 seconds, Figure 2). The amount of time spent on the informed consent page differed by race (*P*=.02) and education (*P*=.01). Time on consent was significantly correlated with consent comprehension score in all consent groups (all *P*<.001; Figure 2).

**Table 2:** Time spent on informed consent and consent comprehension scores among men who have sex with men in a randomized trial of informed consent methods, United States, 2014.

Characteristic		Time spent on consent^a^				Consent comprehension score					
									Post-hoc Tukey comparisons^f^		
		n	Median	IQR^b^	*P* value^c^	n	Mean (SD)^d^	ANOVA^e^* P* value	Mean difference	95% CI	*P* value
All		660	60.5	17.0-162.0		665	6.3 (2.9)				
**Consent group**					<.001			<.001			
	Standard	160	37.0	14.5-88.0		161	5.5 (2.7)		Reference^h^		
	FAQ^g^	178	20.5	11.0-82.0		180	5.2 (2.7)		−0.30	−1.06 to 0.47	.75	
	Professional video	177	115.0	37.0-208.0		178	7.3 (2.8)		1.79	1.02 to 2.55	<.001
	Staff video	145	117.0	39.0-212.0		146	7.3 (2.8)		1.79	0.99 to 2.59	<.001
**Race or ethnicity**					.02			<.001			
	Asian	24	71.0	18.0-212.0		24	4.9 (2.8)		−1.77	−3.47 to −0.07	.04	
	Black	22	95.5	30.0-200.0		22	7.1 (3.0)		0.44	−1.33 to 2.22	.98
	Hispanic	147	25.0	13.0-146.0		147	5.5 (2.4)		−1.19	−1.97 to −0.42	<.001
	Multiracial	26	67.0	24.0-201.0		26	6.4 (2.4)		−0.22	−1.86 to 1.41	.99
	American Indian or Alaska Native	8	27.5	17.0-64.5		8	3.1 (1.6)		−3.52	−6.41 to −0.63	.01
	White	425	68.0	20.0-166.0		430	6.7 (3.0)		Reference		
**Highest level of education**					.008			.001			
	≤ High school	88	39.0	14.0-103.5		88	5.5 (2.0)		Reference		
	Some college	287	53.0	15.0-154.0		288	6.2 (3.0)		0.63	−0.26 to 1.53	.27
	Bachelor's degree	180	65.0	18.0-173.0		183	6.4 (3.0)		0.85	−0.10 to 1.81	.10
	Graduate or professional	103	93.0	37.0-205.0		104	7.1 (3.0)		1.61	0.55 to 2.68	.01
**Age, in years**					.14			.04			
	18-22	164	52.5	16.0-157.0		164	6.1 (2.5)		Reference		
	23-27	234	71.0	21.0-168.0		236	6.7 (3.0)		0.57	−0.12 to 1.26	.13
	28-34	262	53.5	15.0-165.0		265	6.1 (3.0)		−0.03	−0.70 to 0.64	.99

^a^In seconds.

^b^IQR: interquartile range.

^c^Calculated using Kruskal-Wallis tests with alpha=.05.

^d^SD: standard deviation.

^e^ANOVA: analysis of variance.

^f^The null value for mean difference is 0.0.

^g^FAQ: frequently asked questions.

^h^Reference: Reference category for all mean difference comparisons within each characteristic.

Participants in each consent group recorded the following mean comprehension scores: professional video, 7.3 (SD 2.8, range: 1-13); staff-produced video, 7.3 (SD 2.8, range: 1-13); FAQ, 5.2 (SD 2.7, range: 0-13); and standard consent, 5.5 (SD 2.7, range: 1-13; Figure 3). The average consent comprehension score differed significantly by consent group (*P*<.001), age (*P*=.04), race or ethnicity (*P*<.001), and highest level of education (*P*=.001; Table 2). On average, participants in the professional video group scored 1.79 (95% CI 1.02-2.55) points higher than participants in the standard consent group. Similarity, the average score of participants who were in the staff-produced video group was 1.79 (95% CI 0.99-2.59) points higher than the standard consent group. There was not a significant difference in the average scores of participants in the FAQ and standard consent groups (*P*=.75). The number of correct responses significantly differed by consent group for 11 of the 15 consent comprehension questions (Multimedia Appendix 5).

From the mediation analysis, the direct effects of both the professional video (0.93, 95% CI 0.39-1.48) and the staff-produced video (0.99, 95% CI 0.42-1.56) were statistically significant when time spent on consent was held constant. Both video consent methods (relative to standard consent) also indirectly influence consent comprehension score through time spent on consent (professional video 95% CI 0.57-1.16, staff-produced video 95% CI 0.55-1.10; Table 3). This indicates that time on consent functions as a partial mediator of the effect of consent group on consent comprehension score.

**Table 3:** Results of a mediation analysis of time spent on consent in the relationship between consent type and consent comprehension score among 665 men who have sex with men in a randomized trial of informed consent methods, United States, 2014.

Consent group	Total effect			Direct effect			Relative indirect effect through time spent on consent		
	Coefficient^a^	SE^e^	95% CI	Coefficient^b^	SE	95% CI	Coefficient^c^	Boot SE	BC^d^ bootstrap 95% CI
FAQ^f^	−0.32	0.30	−0.91 to 0.26	−0.27	0.27	−0.80 to 0.25	−0.05	0.11	−0.27 to 0.16
Professional video	1.78	0.30	1.19 to 2.36	.93	0.28	0.39 to 1.48	.85	0.15	0.57 to 1.16
Staff video	1.80	0.31	1.19 to 2.42	.99	0.29	0.42 to 1.56	.81	0.14	0.55 to 1.10

^a^Total change in average consent comprehension score.

^b^Change in consent comprehension score when time spent on consent is held constant.

^c^Change in consent comprehension score when consent group is held constant and time spent on consent changes by the amount it differs by consent group.

^d^BC: bias-corrected; number of bootstrap resamples=10,000.

^e^SE: standard error.

^f^FAQ: frequently asked questions.

**Figure 3:**
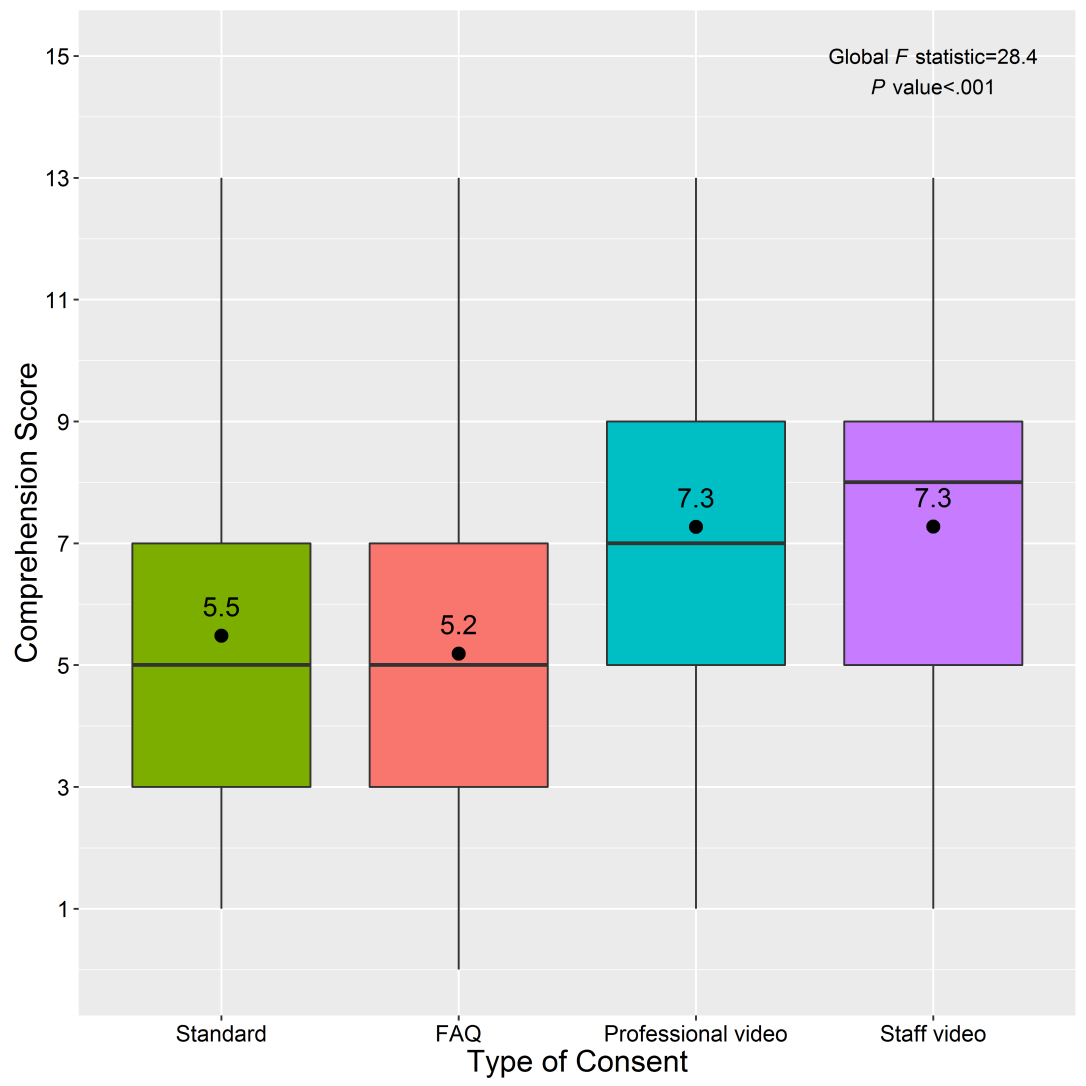
Boxplot of consent comprehension scores, by type of informed consent, among 665 men who have sex with men in a randomized trial of informed consent methods, United States, 2014.

## Discussion

### Principal Findings

The results of our study highlight the need to improve consent comprehension in Web-based HIV research among MSM. On average, participants were missing one-half (video consent group) to two-thirds (standard consent group) of the informed consent information deemed essential for understanding their participation in it. In particular, questions that focused on personal health information, contacts for questions about the study, or HIPAA authorization were answered correctly by less than half of the participants in every group (Multimedia Appendix 5).

Our study also indicates that video-based methods of administering informed consent can lead to increased consent comprehension (compared with traditional consent forms) in Web-based research. Similar improvements in consent comprehension have been seen when video-based consent is used in clinical and surgical settings [32]. The 4 questions that did not see a difference in consent comprehension focused on benefits of participating (“What benefit can you or others reasonably expect from this research?”), voluntary participation (“True or false: Participation in this study is voluntary”), questions about the study (“Who can you contact if you have questions, concerns or complaints about the study?”), and revoking HIPAA authorization (“What will happen if you revoke your authorization?”). The latter 2 questions were incorrectly answered by the majority of participants in each consent group. Researchers may need to provide these questions early in the consent process or find other ways to highlight this information.

Although the median amount of time spent on consent was much higher in the 2 video groups, many participants still passed through the consent page quickly. About half of the participants in the FAQ group spent less than 20.5 seconds on the consent page, indicating many people read very quickly or did not read every question. Whereas the videos may have held the attention of participants longer, half of the participants in the video group spent less than 2 minutes on the consent page, even though the videos were 3.5 minutes long. It is not clear why time spent on consent varied by race or ethnicity.

Comprehension test scores were almost identical between the 2 video groups, indicating a professionally produced video does not provide additional understanding of informed consent over a staff-produced video. The FAQ group did not demonstrate higher consent comprehension compared with the standard group. This could be because the FAQ consent page still required participants to click on each question and read several lines of text, thus making it less accessible to participants who have lower levels of literacy or susceptible to respondents who click on each question but don’t read each one. In addition, the FAQ consent page did not prohibit users from “clicking through” to the survey, even if they did not open any of the FAQ topics.

Time spent on the consent page was significantly correlated with consent comprehension across all groups. Although the correlation coefficient was largest among participants in the FAQ group, comprehension improved as time spent on the consent page (and, presumably, engagement with the consent materials) increased in all groups. The results from the mediation analysis indicate that time on consent does not entirely explain the association between consent group and consent comprehension, which means participants may understand the content in the video better than other methods. Our survey design did not prevent participants from clicking through to the next page before the entire video had finished, and a large number of participants in each group remained on the video page for a period of time that was shorter than the duration of the video. To ensure that all participants are presented the necessary consent information, researchers could design a survey that prevents the user from advancing to the next page until after the video has finished.

### Limitations

There are several limitations to consider when interpreting these results. First, with all Web-based research, decisions made during the deduplication process can lead to biased results [28,33]. As a result of possible fraudulent activity, we had to exclude a large proportion of surveys from this analysis to be most conservative. Though other Web-based research studies that offer incentives have reported similar levels of potentially duplicate or fraudulent surveys, this was uncharacteristically high for many other Web-based research studies with MSM that use a similar deduplication process [33-35]. It is possible our process excluded legitimate responses or failed to exclude some fraudulent responses which may bias findings.

Second, we tested comprehension of consent materials using a survey only among respondents who provided their consent to participate in the main study. We don’t know how many legitimate respondents clicked on the advertisement, encountered the consent materials, and then declined to participate. Although the data would allow us to determine the number of survey responses that landed on the consent page and the number that agreed to participate by each consent randomization group, those data include artificial and duplicate responses. It is possible that one of the consent methods was more likely to discourage participation in the study and if this behavior was also associated with consent, comprehension could lead to biased results. Similarly, all participants had the opportunity to download a PDF version of the written consent and we were not able to track which participants did that. It is possible, though unlikely, that participants in some groups were more likely to download the PDF version. Future implementation of alternative consent methods will also include this option in order to ensure that guidelines for the documentation of informed consent are met [27].

Recruitment for this study targeted social media users and this convenience sample may not be representative of the general community of MSM. However, this type of recruitment is common in research among MSM and allows researchers to collect behavior data for a large number of MSM in a short period of time [36]. Finally, our sample is limited to MSM who report living within the United States, which limits the generalizability of these results to other groups or cultures affected by the HIV epidemic. Although some Web-based HIV risk and prevention research has been conducted in low or middle income countries [37,38], there are additional ethical concerns that must be considered when designing informed consent procedures in these settings [39,40].

Despite these limitations, this research advances the development of innovative methods of administering informed consent in Web-based HIV prevention research among MSM. Although our study tested consent comprehension for a comparative analysis, researchers might also include a Web-based quiz of consent information and require correct answers before proceeding to the actual research [22]. Web-based HIV prevention researchers can also adapt the “teaching then testing” methods used in drug or alcohol abuse research [41].

### Future Research

Future research should continue to improve on these innovative consent procedures and ensure they are optimized for use on mobile phone apps. About 64% of American adults now own a mobile phone that can access the Internet [42] and there is indication that MSM continue to adopt mobile phone technology faster than the general population [43] and are increasingly seeking sexual partners on mobile phone apps [7]. Social media mobile sites and mobile phone apps will continue to be a source of recruitment for HIV prevention research. In the current environment, using videos to administer informed consent are likely to be more effective than traditional, text-based approaches. People are currently used to interacting and receiving information through Web-based videos and matching research processes to these user experiences can improve how we conduct key activities. However, the continued use of studies similar to this one will help determine best practices as they arise and develop in this changing environment.

## Appendix

Multimedia Appendix 1 Screenshot of frequently asked questions (FAQ) consent form used in a randomized trial of informed consent methods, United States, 2014.

Multimedia Appendix 2 Staff-produced video used in a randomized trial of informed consent methods, United States, 2014.

Multimedia Appendix 3 Professional video used in a randomized trial of informed consent methods, United States, 2014.

Multimedia Appendix 4 Consent comprehension questions used in a randomized trial of informed consent methods, United States, 2014.

Multimedia Appendix 5 Number of correct responses to each consent comprehension question, by type of informed consent, in a randomized trial of informed consent methods, United States, 2014.

## Abbreviations

CAPTCHA: Completely Automated Public Turing test to tell Computers and Humans Apart
FAQ: frequently asked questions
HIV: human immunodeficiency virus
HIPAA: Health Insurance Portability and Accountability Act
IP: internet protocol
IQR: interquartile range
MSM: men who have sex with men
PDF: portable document format
STI: sexually transmitted infection
