# Supplementary figures and images for: Use of Videos Improves Informed Consent Comprehension in Web-Based Surveys Among Internet-Using Men Who Have Sex With Men: A Randomized Controlled Trial
Source: J Med Internet Res. 2017 Mar 6;19(3):e64. doi: 10.2196/jmir.6710 (PMC5359419; doi:10.2196/jmir.6710)

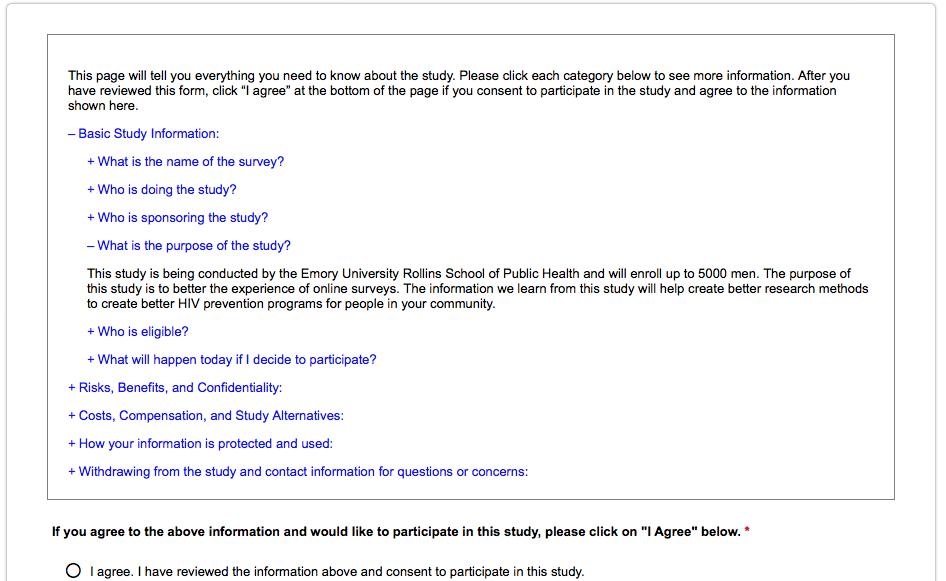

Supplement: Multimedia Appendix 1 [file jmir_v19i3e64_app1.png]
