# Supplementary material for: Use of Videos Improves Informed Consent Comprehension in Web-Based Surveys Among Internet-Using Men Who Have Sex With Men: A Randomized Controlled Trial
Source: J Med Internet Res. 2017 Mar 6;19(3):e64. doi: 10.2196/jmir.6710 (PMC5359419; doi:10.2196/jmir.6710)
Supplement: Multimedia Appendix 4 [file jmir_v19i3e64_app4.pdf]

Multimedia Appendix 4. Consent comprehension questions used in a randomized trial of informed consent methods, United States, 2014.

STUDY DETAILS

1. What is the purpose of this research?
    a. To learn more about the sexual behaviors of men who have sex with men, especially the use of online hookup websites (like A4A, Jack'd, Grindr, etc.)
    b. **To better the experience of online surveys and to create better research methods to create better HIV prevention programs.**
    c. To learn what men think about being in an online HIV research study and how to improve online surveys
    d. To learn more about the best ways to help men avoid sexually transmitted infections through online methods
    e. Don't know
2. Who is eligible for this study?
    a. **Men who are over 18 who use the internet**
    b. Men who have sex with men and women who have recently been tested for HIV
    c. Men who have participated in an online study in the last 12 months
    d. Men who are over 18 years of age and who have recently been tested for HIV
    e. Don't know
3. Who is the Principal Investigator of the study?
    a. Patricia Shapiro, MPH, PhD from Emory University in Atlanta
    b. Phram Surawit, DVM, PhD from Emory University in Atlanta
    c. **Patrick Sullivan, DVM, PhD from Emory University in Atlanta**
    d. Paul Sullivan, MPH, PhD from Georgia Tech University in Atlanta
    e. Don't know

FUNDING

4. Who is sponsoring the study?
    a. The Elton John Foundation
    b. The Kellogg Foundation
    c. The Center for Disease Control and Prevention
    d. **The National Institutes of Health**
    e. The Bill and Melinda Gates Foundation

BENEFITS

5. What benefit can you or others reasonably expect from this research? (Check all that apply)
    a. **Researchers will learn new things to design better online HIV studies to improve HIV testing and prevention programs.**
    b. You will have an opportunity to ask sensitive health questions to a trained medical practitioner.
    c. You will be given personalized services based on your answers from an outside counselor or therapist.
    d. Researchers can learn more about how HIV is transmitted.

INCENTIVES

6. How will you be compensated for your participation in this study?
    a. A wire-transfer of $45 dollars to your personal bank account.
    b. **A $20 Amazon.com gift-card that will be emailed to you.**
    c. $15 in cash that will be mailed to your home address.
    d. A $25 Starbucks gift certificate that will be texted to your phone.
    e. There is no compensation for this study.

RISKS/DISCOMFORTS

7. What are some risks or discomforts you may experience during the research study?
    a. Your contact information will be shared with advertisers
    b. **Some questions, especially those about sex and drugs, might make you uncomfortable.**
    c. Your de-identified information on HIV and STI testing history will be shared with your local health department.
    d. Your answers and identifying information will be shared publically for comparison purposes.
    e. Don't know

CONFIDENTIALITY

8. What personal information will researchers collect about me? (select all that apply)
    a. Name
    b. **IP Address**
    c. Email address
    d. **Age**
    e. **Facebook ID**
9. What will this identifying information be used for? (Check all that apply)
    a. **To send you your incentive.**
    b. To be used for federal government files.
    c. To sell to advertisers and mailing lists for research study funding.
    d. **To make sure you don't take the survey more than once.**
    e. Don't know
10. Who will have access to your health information?
    a. **The principal investigator**
    b. **The research staff**
    c. **Organizations that help conduct the research study**
    d. **Government agencies and University units who make sure the study is being conducted correctly and safely**

ALTERNATIVES

11. True or false: Participation in this study is voluntary.
    a. **True**
    b. False
    c. Don't know

CONTACT INFORMATION

12. Who can you contact if you have questions, concerns or complaints about the study?
    a. **Patrick Sullivan, at EMAIL@emory.edu**
    b. The Consumer Protection Board at consumerprotection@cpb.gov
    c. The National Institutes of Health Helpline at nihhelp@NIH.gov
    d. **The Institutional Review Board at irb@emory.edu**
    e. Don't know
13. Who should you contact if you have questions about your rights as a research subject?
    a. Emory University's Center for Ethics at 404-712-5555 or EUCFE@emory.edu
    b. **The Institutional Review Board at irb@emory.edu**
    c. Phram Surawit at psurawit@emory.edu
    d. The National Institutes of Health Helpline at 301-575-5432 or NIHHELP@NIH.gov
    e. Don't know

WITHDRAWAL FROM THE STUDY

14. How do you revoke your Authorization?
    a. By writing to the Institutional Review Board at irb@emory.edu
    b. **By writing to Patrick Sullivan at EMAIL@emory.edu**
    c. By writing to the HIPAA Control Board @ controlboard@HIPAA.gov
    d. By writing to the Consumer Protection Agency at consumerprotection@cpb.gov.
    e. You cannot revoke your Authorization.

CONSEQUENCES OF WITHDRAWAL

15. What will happen if you revoke your Authorization?
    a. Researchers will not collect any more health information that identifies you, but will continue to use your information in the study.
    b. **Researchers will not collect any more health information from you, but they may keep some identifiable information to maintain the integrity of the study.**
    c. Researchers will delete all your identifiable information and your responses to the survey.
    d. Researchers will continue to use your health information and will keep your identifiable information to report to authorities.
    e. You cannot revoke your Authorization.
    f. Don't know
