# Supplementary material for: Use of Videos Improves Informed Consent Comprehension in Web-Based Surveys Among Internet-Using Men Who Have Sex With Men: A Randomized Controlled Trial
Source: J Med Internet Res. 2017 Mar 6;19(3):e64. doi: 10.2196/jmir.6710 (PMC5359419; doi:10.2196/jmir.6710)
Supplement: Multimedia Appendix 5 [file jmir_v19i3e64_app5.pdf]

Multimedia Appendix 5. Number of correct responses to each consent comprehension question, by type of informed consent, in a randomized trial of informed consent methods, United States, 2014.

	Standard		FAQ		Professional Video		Staff Video		
	n=161		n=180		n=178		n=146		
**Question**	**n**	**%**	**n**	**%**	**n**	**%**	**n**	**%**	***P* value**
What is the purpose of this research study?	88	54.7	72	40.0	93	52.3	88	60.3	0.002
Which of the following best describes who is eligible to participate in this study?	113	70.2	126	70.0	148	83.2	113	77.4	0.01
Who is the Principal Investigator of the study?	65	40.4	68	37.8	104	58.4	85	58.2	<.001
Who is sponsoring the study?	37	23.0	55	30.6	97	54.5	82	56.2	<.001
What benefit can you or others reasonably expect from this research?	82	50.9	89	49.4	91	51.1	81	55.5	0.74
How will you be compensated for your participation in this study?	106	65.8	113	62.8	157	88.2	127	87.0	<.001
What are some risks or discomforts you may experience during the research study?	82	50.9	84	46.7	123	69.1	96	65.8	<.001
What personal information will researchers collect about me?	30	18.6	29	16.1	77	43.3	63	43.2	<.001
What will this identifying information be used for?	32	19.9	27	15.0	60	33.7	53	36.3	<.001
Who will have access to your health information?	6	3.7	5	2.8	19	10.7	13	8.9	0.01
True or false: Participation in this study is voluntary	156	96.9	169	93.9	168	94.4	139	95.2	0.60
Who can you contact if you have questions, concerns or complaints about the study?	9	5.6	12	6.7	21	11.8	9	6.2	0.11
Who should you contact if you have questions about your rights as a research subject?	18	11.2	27	15.0	58	32.6	42	28.8	<.001
How do you revoke your HIPAA Authorization?	33	20.5	35	19.4	50	28.1	48	32.9	0.02
What will happen if you revoke your Authorization?	26	16.2	23	12.8	28	15.7	23	15.8	0.80
